# Haemorrhagic Safety Update of CLEAR-PATH: 30 Day and 12 Month Antiplatelet Therapy After Peripheral Angioplasty

**DOI:** 10.1016/j.ejvsvf.2025.11.005

**Published:** 2025-11-28

**Authors:** Emilien C.J. Wegerif, Manon I. Generaal, Linde J. Vijn, Rutger M. van den Bor, Peter M. van de Ven, Michiel L. Bots, Gert J. de Borst, Çağdaş Ünlü

**Affiliations:** a University Medical Center Utrecht, Division of Vascular Surgery, Utrecht, the Netherlands; b NorthWest Hospital group, Division of Vascular Surgery, Alkmaar, the Netherlands; c University Medical Center Utrecht, Utrecht University, Julius Center for Health Sciences and Primary Care, Utrecht, the Netherlands

**Keywords:** Antiplatelet agent, Chronic limb threatening ischaemia, Intermittent claudication, Percutaneous transluminal angioplasty, Peripheral arterial disease, Randomised controlled trial

## Abstract

**Objective:**

Antiplatelet therapy (APT) bleeding risks after endovascular revascularisation (EVR) in peripheral arterial disease (PAD) patients are lacking. Therefore, this Dutch, multicentre, double blind, placebo controlled randomised trial aimed to determine the haemorrhagic safety of APT within 30 days and 12 months after EVR by using data from the ongoing CLEAR-PATH trial.

**Methods:**

Symptomatic PAD patients after successful EVR were randomised to clopidogrel plus placebo or acetylsalicylic acid for 12 months. The primary endpoint was major bleeding (MB) following the TIMI (Thrombolysis in Myocardial Infarction) classification within 12 months. Secondary endpoints included MB within 12 months following ISTH (International Society on Thrombosis and Haemostasis) and BARC (Bleeding Academic Research Consortium) classification, within 30 days following all classifications, and any bleeding. Analyses were blinded for treatment since the CLEAR-PATH is ongoing. It was agreed that APT groups had MB events above 0.0% and that a difference of 3.0% within 12 months was acceptable.

**Results:**

Between August 2022 and January 2024, 470 patients were randomised. Of these, 103 patients were eligible for 12 month analysis, and 440 patients for 30 month analysis. Patients presented with chronic limb threatening ischaemia (32%) or intermittent claudication (68%). MB incidences within 12 months following TIMI, ISTH, and BARC classifications were 1.9% (95% confidence interval 0.5–6.8), 1.0%, and 2.9%, respectively. The maximum difference between APT groups was 2.8%. MB incidences within 30 days following the TIMI, BARC, and ISTH were 0.9%, 0.7%, and 1.4%, respectively. Six of seven events occurred within 30 days, three of seven events were related to vascular procedures, and one event led to cardiovascular death.

**Conclusion:**

CLEAR-PATH is building a unique, large, well phenotyped PAD cohort. The incidence of MB under APT after EVR differed per classification but was overall low; most occurred within the first 30 days and were related to vascular procedures. The APT regimens raised no haemorrhagic safety concerns; therefore, CLEAR-PATH continues per protocol.

**Trial registration:**

NL80009.041.21, www.clinicaltrialsregister.eu.

## Highlights

• This safety analysis shows a major bleeding incidence between 1 – 2.9% 12 months after endovascular revascularisation.
• Most major bleeds were related to vascular procedures.
• Most major bleeds occurred in the 30 days after endovascular revascularisation.
• Use of clopidogrel plus acetylsalicylic acid did not raise a haemorrhagic safety concern after endovascular revascularisation.

## INTRODUCTION

When prescribing antiplatelet therapy (APT) for patients with peripheral arterial disease (PAD) after endovascular revascularisation (EVR), guidelines recommend considering the atherothrombotic risk associated with PAD, the vascular procedure, as well as the bleeding risk of the patient and the APTs.^1^^,^^2^, ^3^, ^4^, ^5^, ^6^ The latter has gained more awareness since the consequences of bleeding have become clearer due to research in the field of coronary artery disease (CAD); researchers have found a strong relationship between the severity of bleeding and risk of death.^7^ More specifically, major bleeding complications are associated with a mortality rate similar to myocardial infarction, with the increased risk of death primarily occurring within 30 days after the bleeding.^7^^,^^8^ Concerning PAD, insufficient literature is focusing on bleeding events, leading to a large knowledge gap; there is no validated bleeding classification system, studies have heterogeneous designs, the PAD patient mortality rate from a (major) bleeding event is unknown, common origins of bleeding events are unknown, and the APT bleeding incidence is underreported.^1^^,^^9^

The CLEAR-PATH trial was initiated to compare the efficacy and safety of clopidogrel monotherapy with clopidogrel plus acetylsalicylic acid (ASA) within 12 months after EVR.^10^ Due to the limited literature on bleeding events associated with APT following EVR, the safety assessment of the CLEAR-PATH protocol states only: “there is no evidence that dual APT led to a higher risk of major bleeding than mono APT”, based on even fewer and more heterogeneous literature.^11^, ^12^, ^13^, ^14^ Due to this uncertainty, additional data regarding safety of APT is warranted. For this reason, this paper presents the composition of the current CLEAR-PATH cohort, major bleeding events within 30 days and 12 months of APT use after EVR, and the location of major bleeding and its consequences, based on blinded data in the first year of enrolment.

## METHODS

### Trial safety objectives and endpoints

The primary safety objective was to compare rates of major bleeding between clopidogrel monotherapy and clopidogrel plus ASA follosing the Thrombolysis (Table 1).^10^^,^^15^ The TIMI classification was chosen as the primary safety outcome since this is the most used bleeding classification within the field of PAD, which makes data feasible for future comparisons.^16^ The secondary safety objectives focused on the difference in major bleeding between clopidogrel plus ASA and clopidogrel monotherapy within 30 days and 12 months following other bleeding classifications: TIMI classification (30 day analysis only), The International Society on Thrombosis and Haemostasis (ISTH) and the Bleeding Academic Research Consortium (BARC) classification.^17^^,^^18^ For the BARC classification, class 3b or higher was considered as major. Another objective was any bleeding following the TIMI classification within 30 days and 12 months. In addition to bleeding classifications, the location and or cause of the bleeding was recorded and noted. Only blinded results were available for these analyses; therefore, no difference between clopidogrel and clopidogrel plus ASA was shown. However, the last objective was to assess whether the difference in 12 month major bleeding incidence between treatment groups remains within the pre-defined acceptable margin of 3.0%, assuming that no treatment group has a 0.0% risk.

**Table 1:** Definitions of major and minor bleeding following the bleeding classification used in this trial. This table provides an overview of the three bleeding classifications used in this safety analysis.

**TIMI classification**

*Major bleeding* 1. Intracranial haemorrhage or a 5 g/dL (i.e., 3.10 mmol/L) decrease in the haemoglobin concentration, or 2. a 15% absolute decrease in the haematocrit. *Minor bleeding* 1. Observed blood loss: 3 g/dL (i.e., 1.86 mmol/L) decrease in the haemoglobin concentration, or 2. 10% decrease in the haematocrit. No observed blood loss: 4 g/dL (i.e., 2.48 mmol/L) decrease in the haemoglobin concentration, or 3. 12% decrease in the haematocrit. *Minimal bleeding* 1. Any clinically overt sign of haemorrhage (including imaging) that is associated with a <3 g/dL (i.e., 1.86 mmol/L) decrease in the haemoglobin concentration, or 2. <9% decrease in the haematocrit.

**ISTH classification**

*Major bleeding* 1. Fatal bleeding or 2. Symptomatic bleeding in a critical organ or location (e.g., intracranial, intraspinal, intra-ocular, retroperitoneal, intra-articular/pericardial, intramuscular including compartment syndrome); 3. Fall in haemoglobin level of at least 20 g/L (1.24 mmol/L) due to bleeding; 4. Bleeding requiring transfusion of two or more units of blood or red cells. *Clinically relevant non-major bleeding* 1. Any sign or symptoms of haemorrhage (e.g., more bleeding than would be expected) that does not meet the criteria of major bleeding following the ISTH criteria but does meet at least one of the following criteria: a. Require medical interventions by a healthcare professional b. Leading to hospitalisation or increased level of care c. Prompting a face to face evaluation

**BARC classification**

• Type 0: no bleeding • Type 1: bleeding that is not actionable and does not cause the patient to seek unscheduled performance of studies, hospitalisation, or treatment by a healthcare professional; may include episodes leading to self discontinuation of medical therapy by the patient without consulting a healthcare professional • Type 2: any overt, actionable sign of haemorrhage (e.g., more bleeding than would be expected for a clinical circumstance, including bleeding found by imaging alone) that does not fit the criteria for type 3, 4, or 5 but does meet at least one of the following criteria: (1) requiring non-surgical medical intervention by a healthcare professional, (2) leading to hospitalisation or increased level of care, or (3) prompting evaluation • Type 3 ○ *Type 3a* ▪ Overt bleeding plus haemoglobin drop of 3 to <5 g/dL∗ (i.e., 3.10 mmol/L) (provided haemoglobin drop is related to bleed) ▪ Any transfusion with overt bleeding ○ *Type 3b* ▪ Overt bleeding plus haemoglobin drop ≥5 g/dL∗ (i.e., 3.10 mmol/L) (provided haemoglobin drop is related to bleed) ▪ Cardiac tamponade ▪ Bleeding requiring surgical intervention for control (excluding dental, nasal, skin, or haemorrhoid) ▪ Bleeding requiring intravenous vasoactive agents ○ *Type 3c* ▪ Intracranial haemorrhage (does not include microbleeds or haemorrhagic transformation, does include intraspinal) ▪ Subcategories confirmed by autopsy or imaging or lumbar puncture ▪ Intraocular bleed compromising vision • Type 4: CABG related bleeding ○ Peri-operative intracranial bleeding within 48 hours ○ Re-operation after closure of sternotomy for the purpose of controlling bleeding ○ Transfusion of ≥5 U whole blood or packed red blood cells within a 48 hour period^†^ ○ Chest tube output ≥2 L within a 24 hour period • *Type 5: fatal bleeding* ○ Type 5a: Probable fatal bleeding; no autopsy or imaging confirmation but clinically suspicious ○ Type 5b: Definite fatal bleeding; overt bleeding or autopsy or imaging confirmation

TIMI = Thrombolysis In Myocardial Infarction; ISTH = The International Society on Thrombosis and Haemostasis; BARC = Bleeding Academic Research Consortium.

### Data monitoring

Since PAD patients are at high risk of serious adverse events,^2^ only trial related adverse events were registered (among which were atherothrombotic events, bleeding events, death, and peptic ulcers).^10^ The Clinical Trial Centre Maastricht, an independent clinical research organisation, monitored all activities as (source) data verification and verification of potentially missed trial related adverse events in samples of patients' records. For significantly missing information, all patients’ records were screened. Secondly, an adjudication committee, consisting of an independent neurologist, cardiologist, and vascular surgeon, audited all reported events.

### Data analysis

The baseline characteristics and (primary and secondary) safety endpoints of all included patients in the first 1.5 year enrolment period of the ongoing CLEAR-PATH trial were summarised by mean with standard deviation (SD). Categorical variables were summarised by counts and percentages. For primary and secondary endpoint(s), counts and percentages were presented for subjects with a randomisation date (a) 12 months and (b) 30 days before the reference date (1 Feb 2024). It was assumed that the risk of major bleeding within 12 months of follow up was higher than 0.0% in both groups (clopidogrel and clopidogrel plus acetylsalicylic acid) and agreed that a difference of up to 3.0% between the two treatment groups was acceptable. Analyses were performed using R version 4.2.2 (R Foundation for Statistical Computing, Vienna, Austria).^19^ Wilson score interval was used to derive confidence intervals.^20^

### Design

The rationale and design of the CLEAR-PATH trial were reported recently.^10^ In brief, the CLEAR-PATH (EudraCT number: 2021-006611-29) is an investigator initiated, randomised, placebo controlled, double blind, multicentre trial in the Netherlands that primarily compares the efficacy and safety of clopidogrel plus ASA with clopidogrel monotherapy after EVR.

Symptomatic PAD patients with planned EVR with or without thrombo-endarterectomy were included (Table 2). The main exclusion criteria were acute limb ischaemia, re-intervention within two months, or an increased risk of bleeding (i.e., coagulopathy, dialysis, liver failure). After successful EVR, patients were randomised in a 1:1 fashion to clopidogrel 75 mg plus placebo daily or clopidogrel 75 mg plus ASA 80 mg daily for up to 12 months. At each follow up (i.e., four to six weeks post-EVR, every six months, and local visits), patients were assessed for trial related endpoints such as major adverse cardiovascular events, major adverse limb events, and bleeding events.

**Table 2:** Inclusion and exclusion criteria for CLEAR-PATH.^10^ This table outlines the pre-defined eligibility criteria used for screening and enrolment. The inclusion criteria reflect the target population of the CLEAR-PATH trial, while the exclusion criteria are focused on ensuring accurate selection and patient safety.

**Inclusion criteria**

**1.** Lesions of the iliac, femoropopliteal, and or below the knee arteries **2.** At least one TASC lesion **3.** Rutherford (1–6) classes with an indication for an endovascular revascularisation **4.** Proficient understanding of the consequences of enrolment by the patient **5.** Written informed consent by the patient **6.** Age ≥45 years And: **7.** Eligibility of lesions for; a. PTA or recanalisation with or without additional stenting based on prevailing guidelines, or **b.** Hybrid procedure with an endarterectomy of the common femoral artery and additional iliac, femoral, or tibial PTA, or **c.** A re-intervention within 2 months due to a phased treatment.

**Exclusion criteria**

**1.** Acute (limb) ischaemia **2.** Reported intolerance or hypersensitivity to the study medications **3.** Use of anticoagulant therapy (DOACs or coumarin) **4.** Use of NSAIDs >2 weeks which cannot be discontinued **5.** Patients unable to understand the consequences of enrolment in the trial **6.** Patients with a re-intervention due to re-stenosis or re-occlusion within 2 months **7.** Patients with a hybrid procedure other than endarterectomy of the common femoral artery such as femoral bypass **8.** Patients with coagulopathy **9.** Patients with a peptic ulcer confirmed by an oesophagogastroduodenoscopy in their medical history **10.** Patients who are pregnant, contemplating pregnancy, or nursing. **11.** Patients requiring dialysis **12.** Patients with liver failure and at least one of the following criteria; **a.** elevated INR value, or **b.** portal hypertension, or **c.** thrombocytopenia <50x10^9^/L, or **d.** INR, portal tension, or platelet count is unknown

TASC = TransAtlantic Inter-Society Consensus Document on Management of Peripheral Arterial Disease; PTA = percutaneous transluminal angioplasty; DOAC = direct acting oral anticoagulant; NSAID = non-steroidal anti-inflammatory drug; INR = International Normalised Ratio.

This trial was based on the intention to treat principle. Based on an estimated primary efficacy endpoint risk of 30% within one year in the clopidogrel monotherapy arm, an absolute risk reduction of 7% in the clopidogrel plus ASA arm, and 90% power, 450 primary efficacy events (1 696 patients) were required.^10^ These estimates were based on the limited literature regarding post-EVR PAD patients.^21^, ^22^, ^23^, ^24^ The end of study will be reached when either 450 primary endpoint events have been observed or all patients have had a primary endpoint event or have completed the 12 month follow up, whichever comes first. After grant approval in August 2021, preparation started in December 2021 (i.e., legal contracts, medication production, local approval in 14 hospitals, etc).^25^ The NedMec medical ethics review committee approved the CLEAR-PATH trial on 27 May 2022. The first enrolment was on 24 August 2022.

## RESULTS

### Baseline characteristics

**Figure 1:**
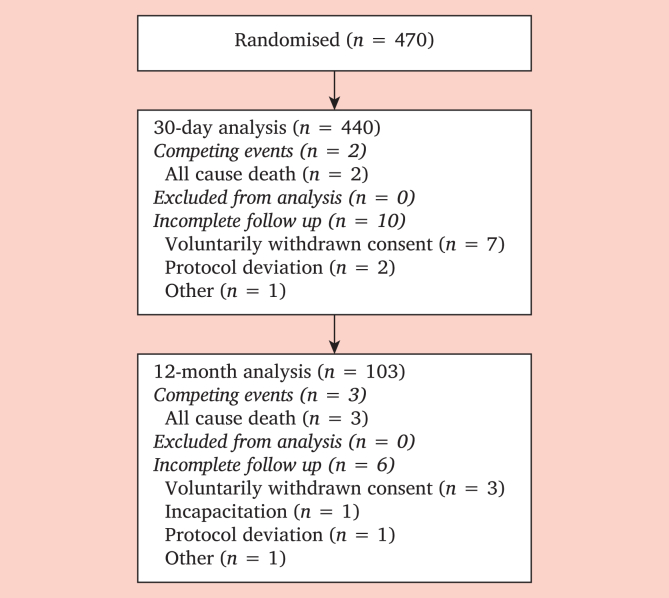
The study flowchart.

From August 2022 to January 2024, 470 patients were randomised across 15 hospitals in the Netherlands. On 31 January 2024, the 12 month follow up results were available for 103 patients, and the 30 day follow up results were available for 440 patients (Fig. 1). Most patients were Caucasian (93%), ex-smokers (55%), and men (66%) with a history of hypertension (72%), hyperlipidaemia (71%), and or intermittent claudication (68%) (Table 3). The mean age was 68 years. Most patients were treated by stent placement (72%) due to stenosis (56%) in the iliac (60%) or femoropopliteal (50%) region.

**Table 3:** The baseline characteristics of the study population containing 470 participants. The characteristics are subdivided into six main categories.

Characteristics	Total (*n* = 470)
*Demographics*	
Mean age – y	68.3 (±8.9)
Female sex	161 (34.4)
Mean body mass index – kg/m^2^	26.3 (±4.4)
*Ethnicity*	
Caucasian	408 (92.7)
Black	6 (1.4)
Asian	7 (1.6)
Multiple, other, or unknown	19 (4.3)
Hyperlipidaemia	331 (71.2)
*Smoking status*	
Never smoked	64 (14.0)
Ex-smoker	251 (54.8)
Current smoker	143 (31.2)
Diabetes mellitus	156 (33.5)
eGFR <60 mL/min/1.73m^2^	65 (14)
Previous coronary artery disease	131 (27.9)
Previous myocardial infarction	62 (13.2)
Previous carotid artery disease	34 (7.4)
Previous stroke	35 (7.4)
Previous heart failure	12 (2.6)
*Peripheral artery disease related history*	
Previous amputation	14 (3.0)
Previous PTA (w or w/o stent)	180 (38.3)
Previous TEA	65 (13.8)
Previous thrombolysis	5 (1.1)
Previous leg revascularisation	193 (41.1)
C*lassification of clinical symptoms*	
Intermittent claudication	311 (67.9)
Chronic limb threatening ischaemia	147 (32.1)
*Ankle brachial index (categories)*	
<0.4	62 (15.9)
0.4–0.69	194 (49.6)
0.7–0.9	94 (24.0)
>0.91 and ≤1.3	40 (10.3)
>1	1 (0.3)
*Type of stenosis*	
Stenosis only	261 (55.5)
Occlusion only	101 (21.5)
Both	31 (6.6)
Endovascular without TEA	393 (83.6)
Endovascular with TEA	72 (15.3)
Iliac region	286 (61.0)
Femoropopliteal region	236 (50.3)
Crural region	48 (10.2)
Multilevel procedure	103 (22.2)
*Stent length*	
No stent	133 (28.3)
Total sum ≤10 mm	172 (36.6)
Total sum ≥10 mm	165 (35.1)
*Medication use*	
Statin	303 (91.5)
Antihypertensive drugs	305 (91.6)

Data are presented as *n* (%) or mean ± SD. SD = standard deviation; eGFR = estimated glomerular filtration rate; PTA = percutaneous transluminal angioplasty; TEA = thromboendarterectomy; mm = millimetre.

### Safety endpoints

The primary safety endpoint occurred in two of 103 (1.9%, 95% Wilson confidence interval [CI] 0.5–6.8) patients (Table 4). Major bleeding within 12 month follow up following BARC and ISTH was found in one (1.0%) and three (2.9%) of a total of 103 patients, respectively. Competing events were registered for three of 103 (2.9%) patients for each classification and concerned only with all cause death. In total, six of 103 (5.8%) patients prematurely ended follow up without any specific type of bleeding for all three classifications. Reasons for premature end of study were loss of follow up, voluntary withdrawal consent, incapacitation, and protocol deviation. With a maximum rate of 2.9% for major bleeding events within 12 months of follow up, and the assumption that the risk of major bleeding events in both groups was greater than 0.0%, the maximum possible difference between the groups was 2.8%.

**Table 4:** Primary and secondary safety endpoints. A. presents data on the number of events of the primary and secondary endpoints, competing events, and incomplete follow up of the 440 participants in the 30 days of follow up group. B. presents data on the number of events of the primary and secondary endpoints, competing events, and incomplete follow up of the 103 participants in the 12 month follow up group.

	A		B	
	*N* (%) patients randomised at least 30 days before reference date (*n* = 440)		*N* (%) patients randomised at least 12 months before reference date (*n* = 103)	
	Safety endpoints			
	Events	Competing event or incomplete FU	Events	Competing event or incomplete FU
Major bleeding following TIMI classification	4 (0.9)	2 (0.5)/10 (2.3)	2 (1.9)	3 (2.9)/6 (5.8)
Major bleeding following BARC classification∗	3 (0.7)	2 (0.5)/10 (2.3)	1 (1.0)	3 (2.9)/6 (5.8)
Major bleeding following ISTH classification	6 (1.4)	2 (0.5)/10 (2.3)	3 (2.9)	3 (2.9)/6 (5.8)
Any bleeding following TIMI classification	12 (2.7)	2 (0.5)/10 (2.3)	7 (6.8)	3 (2.9)/6 (5.8)

TIMI = Thrombolysis In Myocardial Infarction classification; ISTH = The International Society on Thrombosis and Haemostasis classification; BARC = Bleeding Academic Research Consortium classification; FU = follow up.

∗ 3b or higher is considered as major bleeding.

Major bleeding rates within 30 days of follow up following the TIMI, BARC, and ISTH classifications were 0.9% (four of 440), 0.7% (three of 440), and 1.4% (six of 440), respectively (Table 4). The numbers of competing events and incomplete follow ups were low. Any bleeding within 30 days of follow up was found in 12 of 440 (2.7%) patients. Any bleeding within 12 months of follow up following TIMI classification was found in seven of 103 (6.8%) patients. Considering all registered major bleeding events, except for one event (major bleeding due to a gastric ulcer), all major bleeding events happened in the first 30 days and were mostly related to a vascular intervention (Table 5). Two bleeding events were followed by death within 30 days, of which one was a cardiovascular death and one non-cardiovascular death.

**Table 5:** An overview of patients with major bleeding. If a bleeding event was considered major following one of the three bleeding classification, the event is mentioned in the table below. Per major bleeding event, the table shows the timing of the event since the endovascular revascularisation, whether the bleeding event was major or minor following the classification, what the location or cause of the bleeding event was, and the impact of the event.

Patient	12 mo FU analyses	Days after EVR	TIMI	ISTH	BARC∗	Cause and location of bleeding	Impact
P1	Y	6	Major	Major	3b	Haematuria	Hospitalisation; full recovery
P2	N	25	Major	Major	3b	Post-procedural bleeding at procedure site	Prolonged hospitalisation; recovering
P3	N	0	Major	Major	3b	Post-procedural bleeding at procedure site	Cardiovascular death, 13 days after event
P4	N	25	Major	Major	3a	Diverticular haemorrhage	Non-cardiovascular death, 29 days after event
P5	Y	49	Major	Major	3a	Duodenal haemorrhage due to ulcer	Hospitalisation; full recovery
P6	Y	3	Minimal	Major	3a	Haemoglobin fall of unknown aetiology	Temporary extra care via outpatient clinic; recovery unknown
P7	N	0	Minimal	Major	2	Post-procedural bleeding at procedure site	Temporary extra care including additional intervention; recovery unknown

P = patient; mo = months; FU = follow up; EVR = endovascular revascularisation; TIMI = Thrombolysis In Myocardial Infarction classification; ISTH = The International Society on Thrombosis and Haemostasis classification; BARC = Bleeding Academic Research Consortium classification.

∗ 3b or higher is considered as major bleeding.

## DISCUSSION

This safety report contains blinded data from the first 470 patients enrolled in the CLEAR-PATH trial. At baseline, two thirds of the patients had intermittent claudication, and one third had chronic limb threatening ischaemia (CLTI). Based on the current data, approximately 1.9% of patients experienced major bleeding within 12 months after EVR. The major bleeding events occurred with one exception within the first 30 days after EVR and were mostly related to vascular procedures. Despite these data, the start of APT after EVR must not be delayed, given that the risk of atherothrombotic events is significantly higher during this period.^10^ Importantly, with a maximum difference of 2.8% in major bleeding risk within 12 months between dual APT (clopidogrel plus acetylsalicylic acid, DAPT) and mono APT (clopidogrel), the difference is below the pre-specified threshold of 3.0%. These results indicate that APT with either DAPT or single antiplatelet therapy (SAPT) is safe, and therefore, DAPT for up to 12 months does not lead to a clinically relevant increased risk of major bleeding.

The most commonly given antithrombotic therapy for patients with PAD is APT (more specifically, ASA or clopidogrel monotherapy), vitamin K antagonist, and ASA plus low dose rivaroxaban or clopidogrel.^1^^,^^2^, ^3^, ^4^, ^5^, ^6^ A meta-analysis of ATT in PAD patients based on randomised controlled trials (RCTs) showed that for the ATTs described above, an increased risk of major bleeding in patients with vitamin K antagonist and ASA plus low dose rivaroxaban compared with ASA.^16^ The absolute weighted incidences of major bleeding were 2.5% (range 0.0–4.2%, eight RCTs) for ASA, 1.6% (0.00–1.6%, two RCTs) for clopidogrel, 3.4% (range 0.0–8.3%, six RCTs) for ASA plus clopidogrel, 3.6% (0.0–4.3%, three RCTs) for ASA plus low dose rivaroxaban, and 8.1% (one RCT) for vitamin K antagonist (Supplementary Table S1).^16^ However, heterogeneous study designs (i.e., follow up duration and pre- or post-intervention) and different (non-validated) bleeding classifications make definitive conclusions impossible.^16^

Focusing on post-procedural bleeding, the VOYAGER trial found a statistically significantly increased incidence of major bleeding in the subgroup that received ASA plus low dose rivaroxaban (absolute risk of 2.3%) compared with ASA monotherapy (absolute risk of 1.5%) after EVR, following the TIMI classification within 28 months.^26^ The EUCLID trial compared clopidogrel with ticagrelor in symptomatic PAD patients and reported on post-procedural bleeds. EUCLID found a major bleeding incidence of 7.8% and any bleeding incidence of 15.7% within 30 months after EVR, following the ISTH classification.^27^ Comparable findings with CLEAR-PATH were the definition of major bleeding: most experienced a haemoglobin decrease of ≥2 g/dL (1.24 mmol/L) without the need for blood transfusion, and both studies showed low fatal bleeding or bleeding in a critical location (0.4% in EUCLID, 0.0% in CLEAR-PATH). In the studies, approximately half of the post-procedural bleeding events were found in the first seven days, which was comparable with the current findings.

Differences in (non-validated) bleeding classifications must be considered when comparing trials, since they lead to different results (Table 4). The ISTH classification is likely to report the highest incidence of major bleeding since the decrease in haemoglobin level is relatively small.^15^^,^^17^ The BARC classification does not define major bleeding; however, when 3b is considered as major, as in CLEAR-PATH and VOYAGER, the decrease in haemoglobin level corresponds to the TIMI classification.^10^^,^^15^^,^^18^ BARC class 3b or greater contains more components than the TIMI classification and is therefore likely to report higher major bleeding incidences.^15^^,^^18^^,^^26^ Accurate bleeding classification validated for PAD patients is lacking but of interest to predict outcomes and compare APT among studies.

This safety update has a few limitations. The trial was powered for its efficacy endpoint, which did not include major bleeding. Consequently, some relevant factors for bleeding events are missing, such as the sheath size and the vascular closure devices used, which is of interest since most bleeding events found in this analysis were found in the first 30 days. This safety analysis has a substantial proportion of missing data due to competing events and incomplete follow up. Moreover, following the CLEAR-PATH protocol, APT starts one day after EVR; therefore, post-procedural bleeding is not always associated with the APT.^10^

The CLEAR-PATH trial is on target, and the last patient is predicted to be enrolled in 2026 with the trial ending in 2027. Since the start of the CLEAR-PATH in 2023, limited literature has been published on APT in PAD patients following EVR. Moreover, a new guideline has recently affirmed this research gap and highlighted the importance of research in this specific area.^1^

### Conclusion

CLEAR-PATH is an ongoing prospective trial building a unique, large, well phenotyped PAD cohort. The incidence of major bleeding under APT after EVR in PAD patients differed per classification, but was overall low, most seemed to occur in the first 30 days and were related to vascular procedures. The APT regimens used in CLEAR-PATH did not raise haemorrhagic safety concerns; therefore, this study continues per protocol.

## Funding

The CLEAR-10.13039/100005624PATH study is funded by an independent grant provider 10.13039/501100001826ZonMw (dossier number: 80-84800-98-44023).

## Conflict of interest

The authors have no (potential) conflict of interest.
