# Supplementary material for: Haemorrhagic Safety Update of CLEAR-PATH: 30 Day and 12 Month Antiplatelet Therapy After Peripheral Angioplasty
Source: EJVES Vasc Forum. 2025 Nov 28;65:32–9. doi: 10.1016/j.ejvsvf.2025.11.005 (PMC12830239; doi:10.1016/j.ejvsvf.2025.11.005)
Supplement: Multimedia component 1 [file mmc1.pdf]

Supplementary Table S1. Absolute incidence of major bleeding in patients with peripheral arterial disease amongst randomised controlled trials.* This table provides a complete overview of all important trials concerning antithrombotic therapy in patients with peripheral arterial disease based on the data of a recent network meta-analysis. In addition to the network meta-analysis, this table shows the incidence of major bleeding in percentages per trial, per antithrombotic therapy group, and includes a weighted average based on their sample sizes.

		Population	Sample	Major bleeding events	Bleeding classification	Incidence of major bleeding
			**Acetylsalicylic acid monotherapy**			
1	CHARISMA	Symptomatic PAD	1551	27	GUSTO	1.74%
2	COMPASS	Symptomatic PAD	2504	42	Modified ISTH	1.68%
3	WAVE	Symptomatic PAD	1081	24	Own definition	2.22%
4	BOA	PAD + PVI	1324	56	Own definition	4.23%
5	CABBAGE	PAD + PVI	25	0	Own definition	0.00%
6	CASPAR	PAD + PVI	426	5	GUSTO	1.17%
7	Johnson	PAD + PVI	413	15	Own definition	3.63%
8	VOYAGER	PAD + PVI	3278	100	ISTH	3.05%
	Weighted average		10602	269	NA	2.54%
			**Clopidogrel monotherapy**			
1	EUCLID	Symptomatic PAD	6955	109	TIMI	1.57%
2	Li	PAD + PVI	25	0	Own definition	0.00%
	Weighted average		6980	109	NA	1.56%
			**Acetylsalicylic acid plus clopidogrel**			
1	CHARISMA	Symptomatic PAD	1545	26	GUSTO	1.68%
2	CASPAR	PAD + PVI	425	9	GUSTO	2.12%
3	ePAD	PAD + PVI	100	2	TIMI	2.00%
4	Monaco	PAD + PVI	157	13	Own definition	8.28%
5	RIVAL-PAD	PAD + PVI	11	0	TIMI	0.00%
6	PLATO	CAD + PAD	578	46	TIMI	7.96%
	Weighted average		2816	96	NA	3.41%
			**Acetylsalicylic acid plus low-dose rivaroxaban**			
1	COMPASS	Symptomatic PAD	2492	68	Modified ISTH	2.73%
2	RIVAL-PAD	PAD + PVI	9	0	TIMI	0.00%
3	VOYAGER	PAD + PVI	3286	140	ISTH	4.26%
	Weighted average		5787	208	NA	3.59%
			**Vitamin K antagonist (INR 3-4.5)**			
1	BOA	PAD + PVI	1326	108	Own definition	8.14%

PAD = peripheral arterial disease, PVI = peripheral vascular intervention.

*data were retrieved from Willems, LH, et al. (2022)
